# Ants prefer the option they are trained to first

**DOI:** 10.1242/jeb.243984

**Published:** 2022-12-16

**Authors:** Felix B. Oberhauser, Katharina Bogenberger, Tomer J. Czaczkes

**Affiliations:** ^1^ Animal Comparative Economics Laboratory, Department of Zoology and Evolutionary Biology, University of Regensburg, 93053 Regensburg, Germany; ^2^ Centre for the Advanced Study of Collective Behaviour, University of Konstanz, 78464 Konstanz, Germany

**Keywords:** *Lasius niger*, Y-maze, Trail pheromone, Serial position, Primacy, State-dependent learning

## Abstract

The temporal order in which experiences occur can have a profound influence on their salience. Humans and other vertebrates usually memorise the first and last items of a list most readily. Studies on serial position learning in insects, mainly in bees, showed preference for last encountered items. In bees, pheromone presence can also influence motivation, and thus learning. However, neither serial position learning nor the effect of recruitment pheromones on learning have been well investigated in ants. We trained *Lasius niger* ants to make multiple visits to sucrose on a runway which alternated between lemon or rosemary odour, and the presence or absence of trail pheromone, and then tested for preference between the odours on a Y-maze, in order to investigate the effect of pheromone presence on learning. Pheromone presence did not affect ant choice. However, unexpectedly, the ants strongly preferred the first odour encountered. This was explored by the addition of a familiarisation visit without pheromone or odour. The familiarisation visit disabled or reversed this preference for the first odour encountered, with ants now mostly taking their ‘default’ preference by choosing the left side of the maze. Our study found no effect of trail pheromone on learning, but a strong yet fragile preference for the first odour experienced. These different preferences could lead to spatial segregation of foraging activity depending on prior experience and might facilitate efficient resource exploitation by colonies.

## Teaser

**Summary:** Ants preferentially choose odours they encounter first in a serial learning task.

## INTRODUCTION

In a classical study on serial learning, Ebbinghaus (1885) demonstrated that he could best recall nonsense syllables which were either at the start or the end of a list. These effects of serial position (Healy and Bonk, 2008; Murdock, 1962; Nipher, 1878) were referred to as primacy and recency effects (Page and Norris, 1998) and have also been reported in other animals apart from humans (Fagot and Cook, 2006).

In insects, such serial position effects might strongly influence foraging patterns, especially in central place foragers such as bees and ants, where individuals often make repeated sequential visits to multiple food sources. In bees, many studies report recency effects, with recently encountered stimuli not only being remembered better because of more recent experience, but also as a result of retroactive interference of the current stimulus with previously encountered ones (Benard and Giurfa, 2004; Hunt and Chittka, 2015; Menzel, 2009; Prabhu and Cheng, 2008). In contrast, primacy effects require the insect to recall the stimulus that had to be memorised the longest and, to our knowledge, no evidence for primacy effects has been found in social insects so far.

Like bees, black garden ants, *Lasius niger,* obtain most of their calories from honeydew or nectar (Engel et al., 2001) by visiting various food locations, each of which offers characteristic bouquets that are learned by the ants over repeated visits (Czaczkes et al., 2014). Such learned cues are of great importance, and ants will recall previously rewarded locations upon later exposure to such cues (Czaczkes et al., 2014), and will even build up expectations about the taste of a food (Oberhauser and Czaczkes, 2018). However, little is known about serial position effects on foraging decisions in ants.

Apart from serial position effects, learning can be strongly affected by the current internal state. For example, locusts prefer food flavours consumed when hungry to flavours consumed when satiated, even if both are equally nutritious (Pompilio et al., 2006). Such state-dependent learning effects are implicated in seemingly irrational preferences of birds (Kacelnik and Marsh, 2002), bats (Hemingway et al., 2020), rats (Lydall et al., 2010), and ants (Czaczkes et al., 2018) for rewards associated with higher effort.

Finally, learning might also be heavily influenced by context in which cues are encountered (Cheng, 2005). One prominent social context is provided by trail pheromone, an odour trail deposited by many ant species to signal valuable resources (Czaczkes et al., 2015). Thus, ants advertising a good food source might add new contextual information to the area around the food source, and the path to the food, thereby supporting learning. Moreover, by guiding the ants towards a food source, pheromones might reduce contiguity (the time between exposures) between a cue (conditioned stimulus, CS) and the associated food (unconditioned stimulus, US), consequently favouring a learned association (Boakes and Costa, 2014; Menzel, 2009). In honeybees, geraniol, an attractive pheromone, improved olfactory associative learning, while 2-heptanone, an aversive pheromone, impaired learning (Baracchi et al., 2020). Evidence for the effect of trail pheromones on ant learning is, however, mixed (Czaczkes et al., 2016, 2013a), and their impact on foraging ants might be species specific (Oberhauser et al., 2020b; Rossi et al., 2020).

The main aim of this study was to examine whether social context (pheromone presence) affected learning. After finding a striking serial position effect, we expanded the study to investigate the impact of serial position and social context (pheromone) on learning. To this end, we investigated whether ants prefer food that was encountered first or last, and whether a familiarisation visit can disrupt any potential serial position effect. We expected such a disruption since the first exposure to a reward in a new context will be the most surprising, and thus memorable, while on subsequent exposures the presence of a reward is less surprising (Rescorla and Wagner, 1972).

## MATERIALS AND METHODS

### Collection and rearing of colonies

Stock colonies of the black garden ant *Lasius niger* (Linnaeus 1758) were collected on the campus of the University of Regensburg and kept in plastic foraging boxes with a layer of plaster of Paris on the bottom and a circular plaster nest (14 cm diameter, 2 cm high). The collected colonies were queenless and consisted of 500–1000 workers. Queenless colonies forage and lay pheromone trails and are frequently used in foraging and learning experiments (Czaczkes and Kumar, 2020; Detrain et al., 2019). All colonies were kept on a 12 h day:12 h night cycle and were provided *ad libitum* water and 1 mol l^−1^ sucrose solution, supplemented with *Drosophila*. The colonies were deprived of food for 4 days prior to each trial. Tested ants were permanently removed from the colony to prevent pseudo-replication. All applicable international, national and/or institutional guidelines for the care and use of animals were followed.

### Solutions, odours and pheromone extractions

As a reward during the experiment, 1 mol l^−1^ sucrose (Merck KGaA, Darmstadt, Germany) solutions were used. Paper runways were impregnated with lemon or rosemary essential oil (Mit allen 5 Sinnen, Grünwald, Germany) by keeping the runways in an enclosed box containing 100 µl of the corresponding essential oil on filter paper for >2 h (see also Oberhauser et al., 2019).

Trail pheromone was extracted using a modified extraction method from von Thienen et al. (2014). The hindgut of four medium-sized *Lasius niger* workers was ruptured in 1 ml dichloromethane (DCM) solvent to acquire a pheromone strength comparable to that in natural conditions (von Thienen et al., 2014). The extracted pheromone was kept in a freezer between experiments and was exchanged every 4 days. During the experiment, the solutions were kept on ice. Pheromone and DCM was applied onto paper overlays by using a microcapillary tube (Servoprax GmbH) and moving slowly over the paper. This was done 3 times, resulting in ∼6 µl pheromone extract applied to a 9 cm long section of the runway. On each training visit, except for the familiarisation visit (see below), ants encountered scented paper overlays. To prevent potential masking of pheromone by the paper overlay odour, we applied pheromone for the whole 9 cm, the first 6 cm on unscented, the last 3 cm on scented paper overlays. Thus, on the last 3 cm, a combination of odour and pheromone was present leading to the sucrose solution, which was placed on a 1 cm long acetate sheet. After each training visit, the paper overlays as well as the sucrose solution and acetate sheets were exchanged. All colonies were tested between September and November.

### Experimental procedure

On the first training visit, 2–4 ants were allowed onto the setup using a drawbridge. The setup consisted of a 9 cm long runway with a 1 mol l^−1^ sucrose solution at the end. The runway was shielded from visual cues by a 5 cm tall white wall. The first ant to reach the reward was marked with acrylic paint and all other ants were returned to the nest. From now on, only the marked ant was allowed onto the setup via the drawbridge for the remaining visits, which were filmed from above with a Panasonic DMC-FZ1000 camera.

#### Training

On each training visit, the ant encountered either pheromone (pheromone first) or DCM (DCM first) in conjunction with one of the odours (lemon or rosemary) both of which alternated between training visits (e.g. first training visit: DCM+lemon; second training visit: pheromone+rosemary). Thus, each ant underwent one of four possible configurations, which are shown in Table 1. Alternating between odours and DCM and pheromone allowed us to test whether pheromone supports learning, as if it does, we would expect a preference for the odour encounter in the presence of pheromone in a later binary test.

**Table 1.**
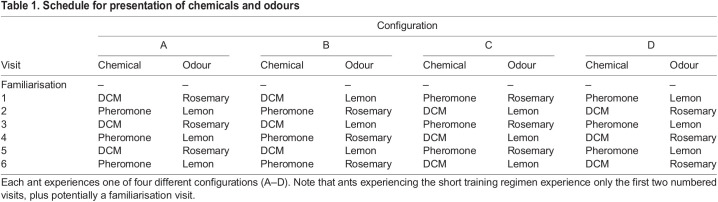
Schedule for presentation of chemicals and odours

Additionally, half the ants underwent an initial familiarisation visit, in which no odour or chemical was presented, to disrupt any potential serial position effect. The pheromone and DCM solutions were code labelled so that the experimenter was blind to the content.

Ants underwent either 2 training visits (one visit to each combination) or 6 training visits (3 visits to each combination). For ants with a familiarisation visit, this resulted in a total of 3 or 7 training visits (familiarisation+training). We varied the number of training visits because we hypothesised that more extensive training would weaken any serial position effects or effects of pheromone on learning. During each training visit to and from the food, we also counted the number of pheromone depositions – a stereotyped behaviour that can be easily quantified in *L. niger* (Beckers et al., 1993) – and the number of U-turns, which are defined as a 180 deg turn followed by walking for at least 2 cm in the new direction.

To measure contiguity, the time between the ant's first encounter of the odour and it touching the sucrose reward was extracted from videos using Solomon Coder software (https://solomon.andraspeter.com/). Contiguity was measured since pheromone could affect learning by reducing the delay between the ant being exposed to an odour (CS) as it steps onto the runway, and it being rewarded by finding the sucrose drop (US), thus improving learning. This is because *Lasius niger* ants run faster on pheromone trails (Czaczkes et al., 2013b).

#### Test

**Fig. 1.**
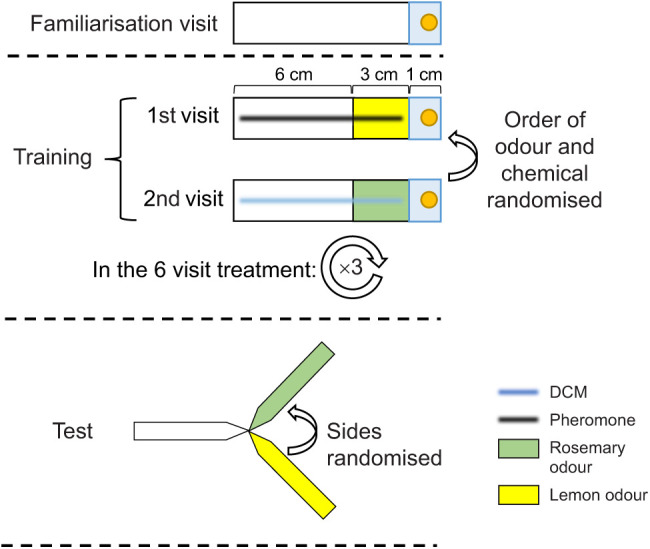
**Experimental procedure.** Half the ants traversed an unscented 9 cm long runway to a receive a 1 mol l^−1^ sucrose reward (yellow dot) in a familiarisation visit (top) and were allowed to return to the nest, before encountering scented and treated runways in the following training visits. The other half immediately started with one of four potential configurations (lemon+pheromone shown here, see Table 1). Pheromone or dichloromethane (DCM) was applied to the whole 9 cm of the runway, while either a lemon or a rosemary scented 3 cm section was placed directly in front of the sucrose solutions. On the next training visit, odours and chemicals were switched. The test was conducted after ants visited both conditions once (in total 2 visits, or 3 visits with familiarisation) or after they visited both conditions three times each (in total 6 visits, or 7 visits with familiarisation). In the test, ants entered a Y-maze in which no reward or pheromone was present, but each arm presented one of the previously encountered odours, the side of which was randomised over ants. The initial decision was scored once the ant crossed a decision line 2 cm inwards of each arm, the final decision once the ant crossed a line 1 cm from the end. At the end of the chosen arm, the ant was allowed on a piece of paper and was gently put back onto the stem until the ant made 5 repeated decisions, which were used as an estimate of persistence and robustness of choice.

After training, the ant was allowed onto a Y-maze (following Czaczkes, 2018). As in training, the Y-maze was surrounded by a 5 cm tall white wall to prevent landmark orientation. An odourless paper overlay was placed on the Y-maze stem, while each arm was scented with either lemon or rosemary odour (see Fig. 1), the side of each odour was assigned pseudo-randomly. Neither pheromone/DCM nor reward was present during the test. Crossing a line on one arm of the maze 2 cm from the bifurcation point was recorded as the initial decision, and crossing a line 2 cm from the end of a maze arm was considered the final decision. Once it reached the end of one arm, the ant was allowed onto a piece of paper and gently placed back to the stem to revisit the maze. This way, we collected 5 test decisions per ant, which provide a measure of choice consistency for each ant. Sample sizes were set pragmatically before data collection began according to the time available for the experiment and the estimated time each trial would take. Altogether, 273 ants were tested.

### Statistical analysis

Data were analysed using generalized linear mixed-effect models (GLMM) (Bolker et al., 2009) in R version 4.1.2 (https://www.r-project.org/). GLMMs were fitted using the glmmTMB package (Brooks et al., 2017 preprint). Since ants from 12 different colonies were tested, we included colony as random intercept in each model. In cases when ants contributed more than one data point (i.e. for pheromone deposition and U-turn data), we also included the ant ID as random intercept. For all models, all possible two-way interactions between the predictors were included. To aid understanding, the model formulas used are shown in the Results section alongside the results.

Each model was tested for fit, dispersion and zero inflation using the DHARMa package (https://CRAN.R-project.org/package=DHARMa). In case of count data, either a Poisson or a negative binomial error distribution was used, depending on the model fit. Model results were obtained using the ANOVA function from the car package (https://CRAN.R-project.org/package=car) and *post hoc* tests were performed with estimated marginal means obtained from the emmeans package (https://CRAN.R-project.org/package=emmeans). For more details, please see the analysis protocol (https://doi.org/10.6084/m9.figshare.20055107.v2). All raw data are shown in Table S1.

## RESULTS

### Test decisions

The model formula of the binomial GLMM was:

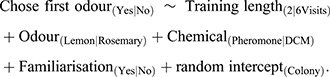


As 16.8% of ants switched Y-maze arm sides after the initial decision, we also ran separate analyses for the final decisions to check for potential differences between initial and final commitment of the ants.

#### Initial decisions

**Fig. 2.**
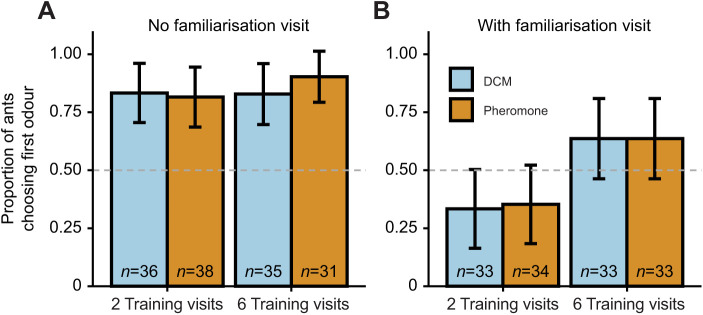
**Proportion of ants preferring the first to the second odour encountered during training in their first test visit.** (A) In training without a familiarisation visit (2 and 6 visits) the majority of ants chose the first encountered odour (2 visits: 86%, *P*<0.001; 6 visits: 87%, *P*<0.001). (B) The addition of a familiarisation visit led to ants choosing the latter odour (2 visits: 31%, *P*=0.028) or randomly (6 visits: 64%, *P*=0.112). Pheromone presence did not affect the ants' choice (χ²=0.26, *P*=0.61) nor did the odour (lemon or rosemary, not shown, χ²=1.1, *P*=0.29). Dashed line indicates chance level; bars indicate mean±95% CI; *n*=number of tested ants.

We found that the ants' odour preference in the test was heavily dependent on the presence or absence of a familiarisation visit during training (χ²=34.9, *P*<0.001, see Fig. 2, compare panels A and B), while pheromone had no significant effect (χ²=0.26, *P*=0.61). Ants also preferentially chose the left side in the Y-maze during the test (χ²=14.9, *P*<0.001), as previously reported (Koch and Czaczkes, 2021). The identity of the odour (lemon or rosemary) did not significantly influence the ants' choices (χ²=1.1, *P*=0.29), nor were any interactions significant (see https://doi.org/10.6084/m9.figshare.20055107.v2).

The preference for the first encountered odour was overall higher for ants with six than two training visits (χ²=8.9, *P*=0.003), which was primarily driven by a tendency to prefer the second encountered odour during the test in treatments with two (but not six) training visits and a familiarisation visit (2 visits: 31%, *P*=0.028; 6 visits: 64%, *P*=0.112, see Fig. 2), thus suggesting a weak recency effect. In treatments without a familiarisation visit, similar preferences were found (2 visits: 86%, *P*<0.001; 6 visits: 87%, *P*<0.001). *Post hoc* tests revealed a tendency in ants with familiarisation visit to primarily go to the first encountered odour during training when it was presented on the left in the maze (left: 65%, *P*=0.102; right: 31%, *P*=0.031), while no such side preference was found in ants without a familiarisation visit (left: 90%, *P*<0.001; right: 80%, *P*<0.001).

#### Repeated initial decisions

When we considered all 5 repeated test decisions of each ant, the presence of a familiarisation visit still heavily influenced the preference for the first encountered odour (χ²=32.7, *P*<0.001, see Fig. S1C) and ants were still more likely to choose the odour presented on the left in the test maze (χ²=9.3, *P*=0.002) while all other predictors did not significantly affect decisions (see https://doi.org/10.6084/m9.figshare.20055107.v2). This time, however, we found a significant interaction between the presence of a familiarisation visit and the position of the first encountered odour in the test maze (χ²=13.4, *P*<0.001). This was caused by the same interesting pattern described above: ants without familiarisation visit went to the first presented odour significantly more often than chance irrespective whether the odour was presented on the left (2 visits: 78%, *P*<0.001; 6 visits: 76%, *P*<0.001) or right (2 visits: 63%, *P*=0.016; 6 visits: 67%, *P*=0.001) during the test. Ants with a prior familiarisation visit, however, mostly chose the first encountered odour when it was on the left during the test (2 visits: 64%, *P*=0.009; 6 visits: 73%, *P*<0.001) but not when it was presented on the right (2 visits: 23%, *P*<0.001; 6 visits (38%, *P*=0.057). In other words, ants which had a familiarisation visit in their training went predominantly to the odour presented on the left during the test instead of preferring the first encountered odour.

#### Final decisions

The final decisions resembled the initial decisions: the presence of a familiarisation visit affected the choice (χ²=15, *P*<0.001, see Fig. S1B) and pheromone had no effect (χ²=3.1, *P*=0.077). We again found an interaction between the position of the first encountered odour in the test maze and the presence of a familiarisation visit (χ²=4.1, *P*=0.042). *Post hoc* tests showed that, as above, ants in treatments without a familiarisation visit had a strong preference for the first odour irrespective of its position in the test maze (left: 84%, *P*<0.001; right: 82%, *P*=0.001), while ants in treatments with a familiarisation visit preferentially chose the odour presented on the left (left: 65%, *P*=0.072; right: 34%, *P*=0.067).

#### Repeated final decisions

Including all 5 test visits per ant yielded similar results. The familiarisation visit strongly affected the choice (χ²=20.8, *P*<0.001, see Fig. S1D), pheromone had no effect (χ²=2.7, *P*=0.099) and ants preferred the left during the test (χ²=4.7, *P*=0.029). Again, we found an interaction between the position of the first encountered odour in the test maze and the presence of a familiarisation visit (χ²=5, *P*=0.026), but this time the preferences in the treatments with a familiarisation visit were very weak (left: 41%, *P*=0.419; right: 57%, *P*=0.351). Additionally, the training length also weakly interacted with the presence of a familiarisation visit (χ²=5.1, *P*=0.024).

### Contiguity

We used a ratio of 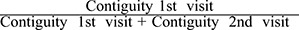 as dependent variable, which means the higher the ratio, the longer the ant spent between CS and US in the first compared with the second training visit. As we only looked at the ratio of the first two visits, we did not include training length in the beta regression model:

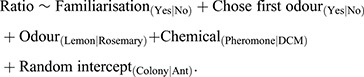


Importantly, we found no significant effect of the choice the ant made in the test on the ratio (χ²=0.4, *P*=0.52). In other words, differences in contiguity (time between CS and US) of the first two training visits could not explain the preference for the first encountered odour. We found an interaction between familiarisation visits and pheromone (χ²=12.1, *P*<0.001), which reflects the fact that ants experienced higher contiguity (spent less time between CS and US) on pheromone than on DCM and overall higher contiguity without prior familiarisation visits – a finding that is consistent with ants making U-turns mostly on the second training visit when no pheromone is present (see ‘U-turns’ section; Fig. 4).

### Pheromone depositions

**Fig. 3.**
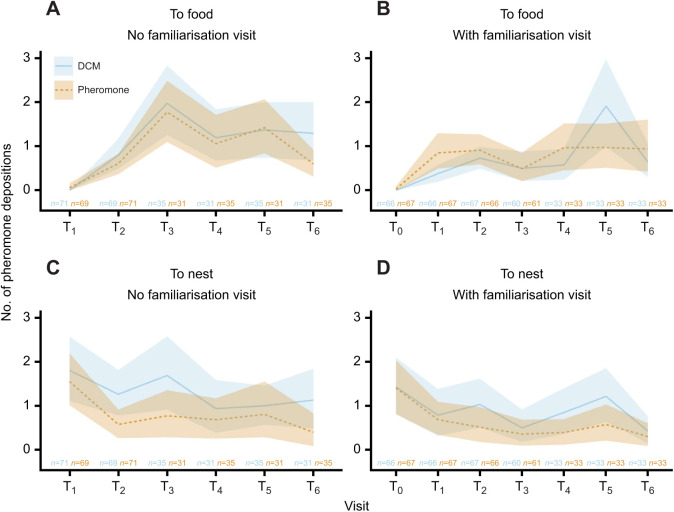
**Number of pheromone depositions on the way to the food or on the way back to the nest of up to 6 training visits (T_1_–T_6_) and the familiarisation visit T_0_.** Depositions on the way to the food (A,B) or on the way back to the nest (C,D) without (A,C) or with (B,D) a familiarisation visit. While the presence of chemicals and familiarisation visits did not affect pheromone depositions on the way to the food (χ²=0.12, *P*=0.728, χ²=0.23, *P*=0.631, respectively), on the way back to the nest, ants deposited less pheromone when it was already present on the runway (χ²=21.9, *P*<0.001) and overall, deposited less following familiarisation visits (χ²=15.9, *P*<0.001). Note that lemon and rosemary odours are pooled in this figure. Line indicates mean; shaded area shows 95% CI; *n*=number of observed pheromone depositions (left: DCM; right: pheromone).

We separately analysed pheromone depositions on the way to the food and back to the nest. To simplify the analysis, we further pooled training regimes according to the presence or absence of a familiarisation visit. To account for variability between training visits, which was not the focus of interest here, we added training visits as an additional random intercept. Familiarisation visits were excluded from the analysis as neither odours nor chemicals were present in these but are shown for completeness in Fig. 3. The formula of the negative binomial model was:

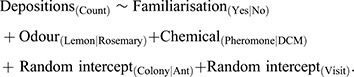


On the way to the food, we observed slightly more depositions on training visits with the lemon odour (χ²=5.8, *P*=0.016), while all the other predictors had no significant effect (*P*>0.19 in all cases, see https://doi.org/10.6084/m9.figshare.20055107.v2).

On the way back to the nest, we found that DCM consistently led to more pheromone depositions (χ²=21.9, *P*<0.001), which indicates that the ants perceived the pheromone and modulated their laying behaviour accordingly (Beckers et al., 1993). Moreover, familiarisation visits also led to overall lower depositions (χ²=15.9, *P*<0.001). Again, the lemon odour led to slightly more depositions (χ²=9.2, *P*<0.001).

### **U**-turns

**Fig. 4.**
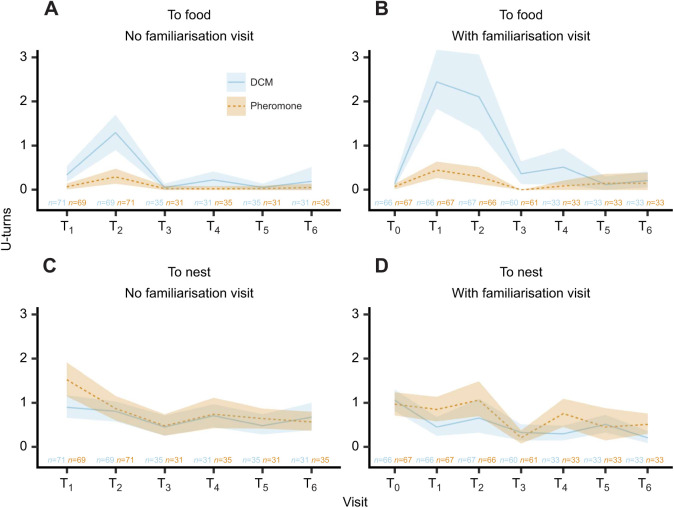
**Average U-turns on the way to the food (top row) or on the way back to the nest (bottom row) of up to 6 training visits (T_1_–T_6_) and familiarisation visits (T_0_).** U-turns on the way to the food (A,B) or on the way back to the nest (C,D) without (A,C) or with (B,D) a familiarisation visit. On the way to the food, ants made more U-turns on runways without pheromone (χ²=202.3, *P*<0.001) and more after familiarisation visits (χ²=37, *P*<0.001). On the way back, we observe the exact opposite, as ants turned more when pheromone was present (χ²=17.4, *P*<0.001) and no familiarisation visit was conducted (χ²=11, *P*<0.001). Note that lemon and rosemary odours were pooled in this figure. Line indicates mean; shaded area shows 95% CI; *n*=number of observed U-turns (left: DCM; right: pheromone).

As with pheromone depositions, we analysed U-turns on the way to the food and back to the nest separately. Again, no familiarisation visits were included but are shown for completeness in Fig. 4. The model formula of the Poisson model used the same predictors as the pheromone model:

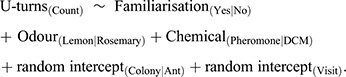


We found that ants made fewer U-turns on the way to the food when pheromone was present (χ²=202.3, *P*<0.001, see Fig. 4), which supports its role in guiding ants. Furthermore, ants made more U-turns when they first made a familiarisation visit (χ²=37, *P*<0.001). This is mostly due to the high number of U-turns on the training visit following the familiarisation, potentially caused by the introduction of the odours and greatly suppressed by the presence of pheromone (see Fig. 4).

The U-turn behaviour on the way back to the nest interestingly revealed an inversion, with pheromone leading to more U-turns overall (χ²=17.4, *P*<0.001). Similarly, treatments with familiarisation visits now had overall fewer U-turns (χ²=11, *P*<0.001).

## DISCUSSION

In our study, we discovered a strong effect of training order on subsequent choice: An ant's decision to visit a location guided by odour was strongly affected by the first encountered odour cue, which contrasts with previous studies in which the last cue dominated the insect's decisions (Benard and Giurfa, 2004; Cheng, 2005; Cheng and Wignall, 2006; Hunt and Chittka, 2015; Menzel, 2009; Prabhu and Cheng, 2008, all on honeybees or bumblebees). In our study, ants consistently preferred the first encountered odour irrespective of pheromone presence, as previously reported for this ant (Wendt et al., 2020). This behaviour is robust to changes in cue and visit number – ants trained first to find food on one side of a Y-maze, and then on a second visit to find food on the opposite arm, choose the first-rewarded location ∼80% of the time (L.-A. Poissonnier, Y. Hartmann and T.J.C., manuscript in preparation). However, in other respects this strong effect is highly fragile: preference is completely lost or even reversed upon introduction of a prior familiarisation visit.

Two non-mutually exclusive mechanisms could explain these findings. Firstly, our observations could be explained by the classic learning model proposed by Rescorla and Wagner (1972), where associative strength is, all else being equal, dependent on the discrepancy between the predicted and encountered stimuli or, in other words, the degree of surprise. Upon first exposure to a new context, the context will add to the associative strength of the first encountered stimulus, thus favouring a primacy effect. When the new context is introduced prior to stimulus presentation, however, it does not add to the predictive power of the first encountered stimulus, thus making the first and second encountered odours equal.

An alternative explanation for our findings is that, after their initial encounter with the food, the ants are less hungry, and thus food becomes less of a motivation to learn in subsequent visits. Such state-dependent learning has been demonstrated in insects in both foraging (Pompilio et al., 2006) and mating (Boehm et al., 2022) contexts, and has been implicated in the driving of preference in ants (Czaczkes et al., 2018). While ants continue to make many tens of foraging visits if allowed, demonstrating a consistent and high motivation (Oberhauser et al., 2020a), the first encounter with a food source after 4 days of food deprivation is likely to be especially salient.

The strong preference for the first encountered stimulus in a series is especially surprising, as it must be stored in memory the longest. The reason why such effects were not reported in insects before might have to do with the experimental procedures: our list of two stimuli represents a minimal and highly tractable scenario for serial learning effects and we presented equal rewards for both odours, thus allowing a clear interpretation of serial position effects. Many studies on serial learning in social insects involve pretraining or familiarisation visits (Hunt and Chittka, 2015; Menzel, 2009; Prabhu and Cheng, 2008) and such prior exposures could remove context effects, which might explain the frequent reports of recency effects.

Interestingly, the addition of a familiarisation visit in our study only led to a weak recency effect after two training visits, which disappeared when taking into account repeated decisions or corrected decisions (final decisions). Instead, in these cases the ants seemed to preferentially choose the left side of the maze. This bias happened despite a shielding wall surrounding the maze and suggests that the ants did not prefer either odour cue in these trials, but instead referred to internal biases guiding their decisions – as was reported before in this species (Oberhauser et al., 2020a). However, in the absence of a shield wall, the bias is towards the visually more cluttered view (Koch and Czaczkes, 2021). The absence of a recency effect and the observation that the primacy effect was of similar strength after 2 and 6 training visits indicates that the initial context of a new route heavily affects the ant's later choices, leading to (at least) short-term preferences for these cues over other, equally rewarding ones.

Unlike the serial position, pheromone did not significantly affect the choices of ants in our study. This suggests that the presence of pheromone does not alter learning in a systematic way, which is in accordance with other studies which found no effect of pheromone on learning in *Linepithema humile* (Rossi et al., 2020) or *Lasius niger* (Oberhauser et al., 2020b; Czaczkes et al., 2016, but see Czaczkes et al., 2013a). At a collective level, such independent evaluation of a food source by multiple ants (conditioned amplification: Feinerman and Korman, 2017) is crucial for keeping positive feedback in check and preventing collective biases during decision making. This is in contrast to honeybees (*Apis mellifera*), in which pheromones with a positive or negative valence do influence appetitive motivation and thus learning (Baracchi et al., 2020). The honeybee recruitment system is less susceptible to positive feedback trapping and bees are much less likely to be exposed to pheromones when foraging.

U-turns on the way to the food were predominantly observed after changes in the setup, usually the second visit in treatments with and without familiarisation visit, which suggests that these U-turns reflect the ant's uncertainty of their route choice. Accordingly, U-turns in these situations were greatly reduced when pheromone was present, although this pattern interestingly reversed on the way back to the nest. Here, ants made more U-turns when pheromone was present, which might indicate that here, the ants tried to memorise the way to the food better by turning around to obtain snap shots of the path to the food (Nicholson et al., 1999). This also explains why the measured contingency – the time between odour (CS) and sucrose (US) exposure – on the way to the food was significantly shorter in trials which had pheromone present. Importantly, this difference in contingency did not systematically influence the preference for the first encountered odour.

The importance of the order of encountered cues might also provide insights to ant foraging: pheromone trails often act as a scaffold for naïve ants to build up a route memory to productive locations (Collett et al., 2003). Naïve ants following a trail might show a preference for the food type or location encountered first, over others encountered later. Ants familiar with the surroundings, in contrast, might not show such preference and instead disperse further away from these advertised food sources.

## Supplementary Material

10.1242/jexbio.243984_sup1 Supplementary information
