# Supplementary material for: Ants prefer the option they are trained to first
Source: J Exp Biol. 2022 Dec 16;225(24):jeb243984. doi: 10.1242/jeb.243984 (PMC10088526; doi:10.1242/jeb.243984)
Supplement: Supplementary information [file jexbio-225-243984-s1.pdf]

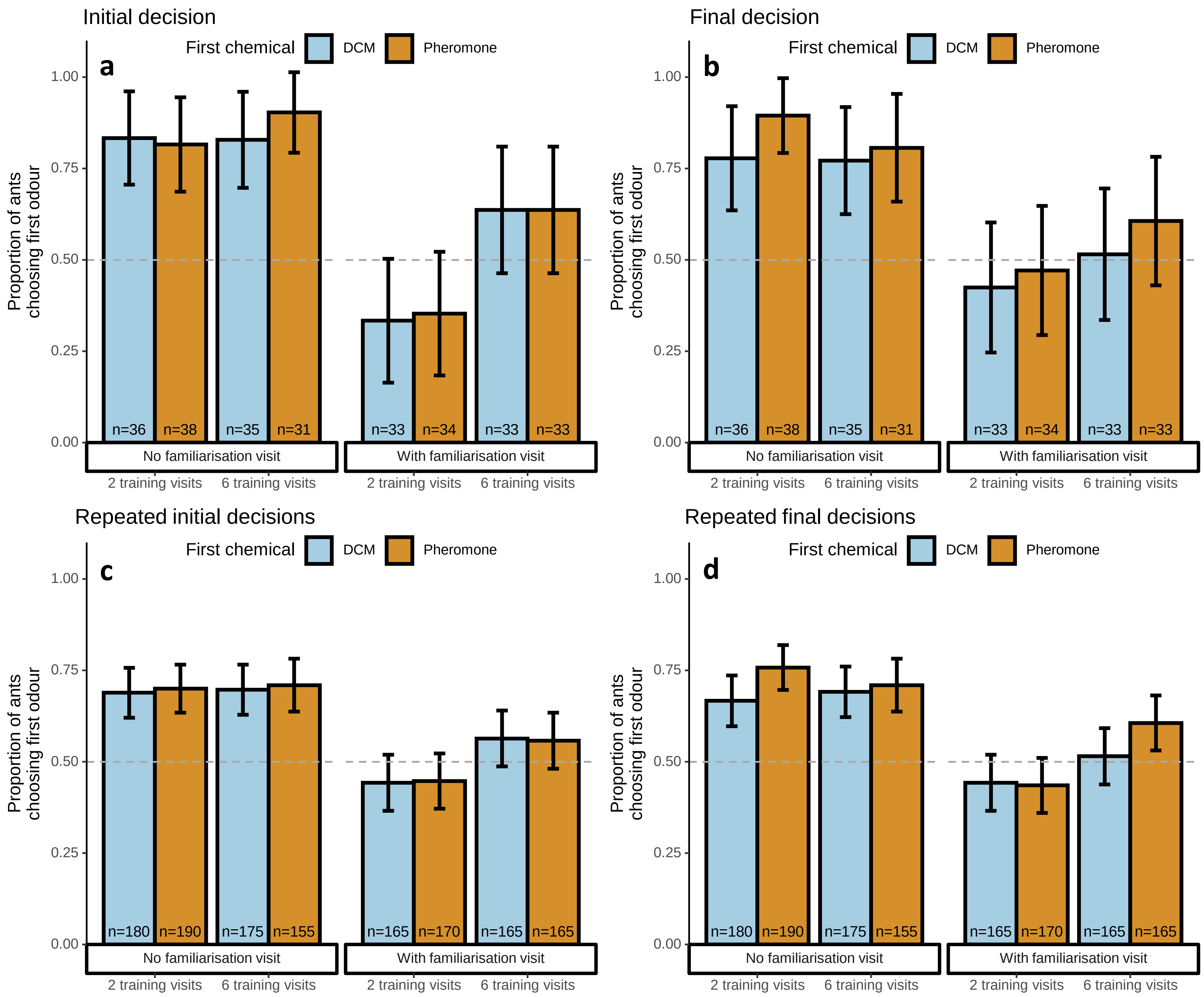

Fig. S1. Overview of the obtained proportions of ants choosing the first odour calculated from initial and final decisions of ants in the test maze with and without repetitions. The initial decision (a) was scored once the ant crossed a decision line 2cm inwards of each maze arm, the final decision (b) once the ant crossed a line 1cm from the end. For repeated decisions (c,d), all five repeated decisions of each ant were included. Dashed line = chance level, bar = mean, error bar = 95% c.i., n = number of tested ants (a,b) or total decisions (c,d).

Table S1.

Click here to download Table S1
